# Assessment of the Effect of *Satureja montana* and *Origanum virens* Essential Oils on *Aspergillus flavus* Growth and Aflatoxin Production at Different Water Activities

**DOI:** 10.3390/toxins12030142

**Published:** 2020-02-25

**Authors:** Marta García-Díaz, Jessica Gil-Serna, Belén Patiño, Esther García-Cela, Naresh Magan, Ángel Medina

**Affiliations:** 1 Department of Genetics, Physiology, and Microbiology, Faculty of Biology, University Complutense of Madrid, Jose Antonio Novais 12, 28040 Madrid, Spain; martga43@ucm.es (M.G.-D.); belenp@ucm.es (B.P.); 2 Applied Mycology Group, Cranfield Soil and AgriFood Institute, Cranfield University, Bedford MK43 0AL, UK; e.garcia-cela@herts.ac.uk (E.G.-C.); n.magan@cranfield.ac.uk (N.M.); 3 Biological and Environmental Sciences, School of Life and Medical Sciences, University of Hertfordshire, Hatfield AL109AB, UK

**Keywords:** Aflatoxin, bioscreen, preservatives, essential oils, food security

## Abstract

Aflatoxin contamination of foodstuffs poses a serious risk to food security, and it is essential to search for new control methods to prevent these toxins entering the food chain. Several essential oils are able to reduce the growth and mycotoxin biosynthesis of toxigenic species, although their efficiency is strongly influenced by the environmental conditions. In this work, the effectiveness of *Satureja montana* and *Origanum virens* essential oils to control *Aspergillus flavus* growth was evaluated under three water activity levels (0.94, 0.96 and 0.98 a_w_) using a Bioscreen C, a rapid in vitro spectrophotometric technique. The aflatoxin concentrations at all conditions tested were determined by HPLC-FLD. *Aspergillus flavus* growth was delayed by both essential oil treatments. However, only *S. montana* essential oil was able to significantly affect aflatoxin production, although the inhibition percentages widely differed among water activities. The most significant reduction was observed at 0.96 a_w_, which is coincident with the conditions in which *A. flavus* reached the highest levels of aflatoxin production. On the contrary, the treatment with *S. montana* essential oil was not effective in significantly reducing aflatoxin production at 0.94 a_w._ Therefore, it is important to study the interaction of the new control compounds with environmental factors before their application in food matrices, and in vitro ecophysiological studies are a good option since they provide accurate and rapid results.

## 1. Introduction

Mycotoxins are fungal secondary metabolites with adverse effects on human and animal health. To date, more than 400 different molecules, produced by several types of fungi, have been characterized [1]. Because of their high toxicity, aflatoxins B_1_, B_2_, G_1_, and G_2_ (AFB_1_, AFB_2_, AFG_1_, and AFG_2_) are the most important, and are produced by the species of *Aspergillus* section *Flavi*, mainly *Aspergillus flavus* [2]. These toxins can contaminate a wide range of agricultural commodities, either in the field or during storage, and they are considered ubiquitous contaminants of the food supply throughout the developing world [3]. AFB_1_ has been described as the most toxic naturally occurring human carcinogen and as the cause of hepatocellular carcinoma in humans and animals [3,4]. The International Agency for Research on Cancer (IARC) has classified the “naturally occurring mixes of aflatoxins” as a Group 1 human carcinogens [5].

Aflatoxin (AF) contamination of feed and food products poses a serious risk to food security and leads to important economic losses due to the impossibility to market contaminated products as well as veterinary and health costs. Most countries have established maximum levels of these contaminants allowed in food products [5]. One of the main problems is that mycotoxins are extremely stable compounds. They are heat-resistant, with melting temperatures above 250 °C, and tolerate a wide pH range, from 3 to 10 [6,7]. Furthermore, mycotoxins have nondetectable sensory characteristics and they do not change the organoleptic properties of food products. Thus, once mycotoxins are present in raw ingredients, they are very difficult to eliminate. Therefore, preventing contamination is the best approach and thus it is essential to establish adequate control methods to prevent AFs from entering the food chain [8]. The best strategy to prevent their presence in agrifood products is to completely avoid fungal growth. However, this might be complicated due to the way food and feed materials are harvested, stored and processed. Moreover, the presence of the fungus is not always associated with the presence of mycotoxins, as the ecological conditions for mycotoxin production are narrower than for fungal growth. Thus, it becomes more important to look for methods that not only focus on controlling fungal growth in food matrices, but also on preventing the synthesis of mycotoxins [6,9].

Many factors influence *A. flavus* growth and AF production in food products, including nutritional composition, temperature, pH, water activity (a_w_), atmospheric composition, and storage times, as well as the presence and concentration of preservatives [10]. The application of food preservatives to control mycotoxin-producing fungi is effective. However, consumers are now demanding safer and more ecofriendly products free from chemicals. In this context, natural plant extracts are considered to be good alternatives [11]

Essential oils (EOs) are aromatic extracts obtained mostly from plant material and have demonstrated strong antimicrobial, antitoxigenic and food preservative properties as well as low toxicity towards animals and humans [12]. They are allowed in food products, have less environmental impact and, therefore, a wider public acceptance [13]. These natural plant extracts are recognized as safe on the GRAS (Generally Recognized As Safe) list, and are used in various sectors, such as agriculture (plant fortifiers, biostimulants, pesticides, postharvest or herbicides), food industry (preservatives or flavorings) and pharmaceutics (aroma compounds or functional ingredients) [13]. In addition, their use is approved for ecological agriculture [14]. Several EOs have been reported to reduce not only the growth of toxigenic fungal species but also to interfere in mycotoxin biosynthesis to some extent [15,16]. *Satureja montana* (SM) and *Origanum virens* (OV) EOs are highly rich in carvacrol and thymol, respectively, which are responsible for their antifungal properties [17].

It is well known that controlling the dose of preservatives is crucial, since suboptimal concentrations could lead to stimulation of both growth and toxin accumulation [18]. The efficacy of EOs is also influenced by the environmental conditions, mainly a_w_ and temperature [19]. Therefore, it is important to unravel the interactions between these environmental factors and antifungal compounds. The study of these interactions required laborious in vitro ecophysiological studies to evaluate fungal growth and mycotoxin production, which requires the use of a lot of material and the planning of long-term experiments. The use of Bioscreen-C Microbiological Growth Analyzer for mycological studies is a quick method to study the effects of multiple factors on mold growth [10,20,21].

The aim of this work was to evaluate the effect of two EOs extracted from SM and OV on the early growth and ability to produce AFs by two *A. flavus* strains (A7 and A10) at several EO concentrations (0, 350, 700, and 1000 μg/mL) and three a_w_ conditions (0.94, 0.96, and 0.98).

## 2. Results

### 2.1. Effect of Satureja montana and Origanum virens Essential Oils under Different Water Activities on Aspergillus flavus Growth

**Figure 1:**
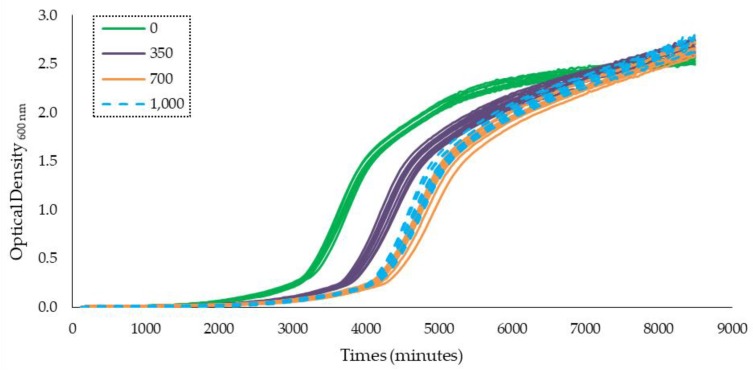
Growth curve obtained using the Bioscreen C analyzer representing optical density at 600 nm for 6 days for *A. flavus* A7 at 0.94 a_w_. Ten replicates for each concentration (0, 350, 700, and 1000 µg/mL) tested are represented. Concentrations of essential oils are represented in the legend.

The growth curves obtained using Bioscreen-C for the two strains of *A. flavus* (A10 and A7), in Yeast Extract Sucrose (YES) medium supplemented by different concentrations (0, 350, 700, and 1000 µg/mL) of *Satureja montana* (SM) and *Origanum virens* (OV) essential oils (EOs), under the three water activities tested (0.94, 0.96, and 0.98 a_w_), are shown in Appendix A. As an example, Figure 1 shows the growth curve of *A. flavus* A7 strain at 0.94 a_w_ with different OV EO concentrations. The growth curves represent the optical density (O.D) units at 600 nm over time.

In this study, comparisons between treatments were done using the time to detection (TTD). TTD is described as the necessary time for fungal growth to reach a specific O.D level with a treatment. Medina et al. (2012) also described a direct relationship between O.D and *A. flavus* biomass [20]. In this work, the TTD was calculated using an O.D at 600 nm = 0.2.

**Figure 2:**
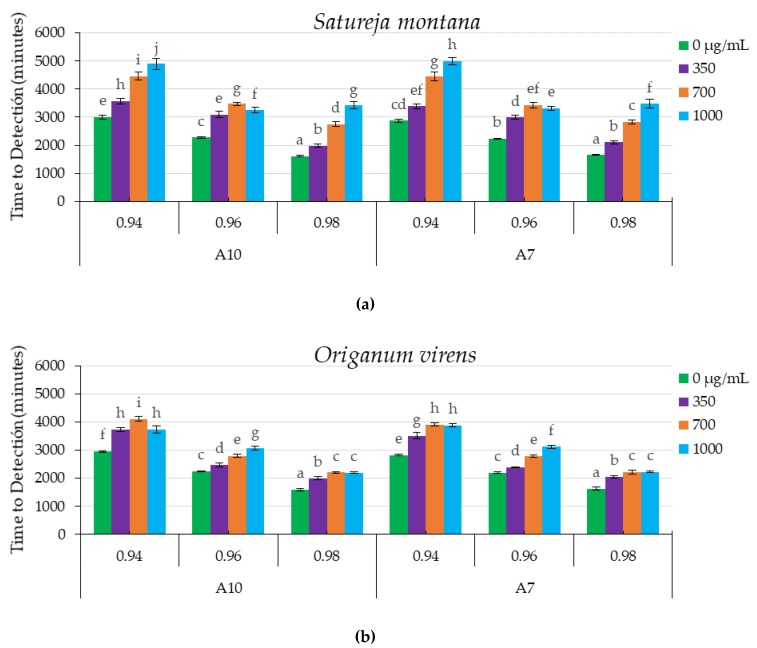
Time to detection (TTD, minutes) at 0.2 nm of Optical Density (O.D) of fungal growth of two *A. flavus* strains (A10 and A7) under different water activity levels (0.98, 0.96 and 0.94 a_w_) at different concentrations (0, 350, 700 and 1000 µg/mL) of *Satureja montana* (**a**) and *Origanum virens* (**b**) essential oils. Values are the means of 10 replicates ± standard errors. Means with a common letter are not significantly different (*p* > 0.05). Concentrations of essential oils are represented in the legend. In all cases statistical analysis was performed independently for each essential oil (EO) and isolate.

Figure 2 shows the TTD for all the combinations of EO concentrations and a_w_ for the two *A. flavus* strains tested.

The statistical analyses regarding the influence of SM EO treatment on the growth of both strains (Figure 2a) showed a significant effect of EO concentrations (*p* < 0.0001), and a_w_ levels (*p* < 0.0001). The interaction between both factors (a_w_ and EO concentration) was also statistically significant (*p* < 0.0001). The highest antifungal properties, related to the less favorable conditions for growth (highest TTD), were obtained with 1000 µg/mL of SM EO and 0.94 a_w_, with approximate TTD values of 4900 and 5000 min in the cases of A7 and A10 strains, respectively. There was a direct relationship between the highest SM EO concentrations and increases in TTD, showing delayed fungal growth. Interestingly, at 0.96 a_w_ and for both *A. flavus* strains, more inhibition was observed at 700 µg/mL than at 1000 µg/mL. In general, the treatment with SM EO retarded fungal growth in relation to the corresponding control at all a_w_ levels tested.

The statistical analyses showed that, for OV EO, the growth of both strains tested (Figure 2b) was significantly affected by the EO concentrations (*p* < 0.0001) and a_w_ levels (*p* < 0.0001). As previously shown, the interactions between both factors were also statistically significant (*p* < 0.0001). The highest antifungal effect was observed at 0.94 a_w_ and 700 µg/mL, with values of approximately 4000 min for both isolates of *A. flavus*. For the A7 strain, there were no significant differences between the two higher doses tested (700 and 1000 µg/mL) of OV EO at 0.94 and 0.98 a_w._ In the case of the A10 strain, the maximum delay in fungal growth was obtained at 0.94 a_w_ and 700 µg/mL. In all a_w_ conditions tested, the application of OV EO delayed fungal growth with respect to the corresponding control.

**Figure 3:**
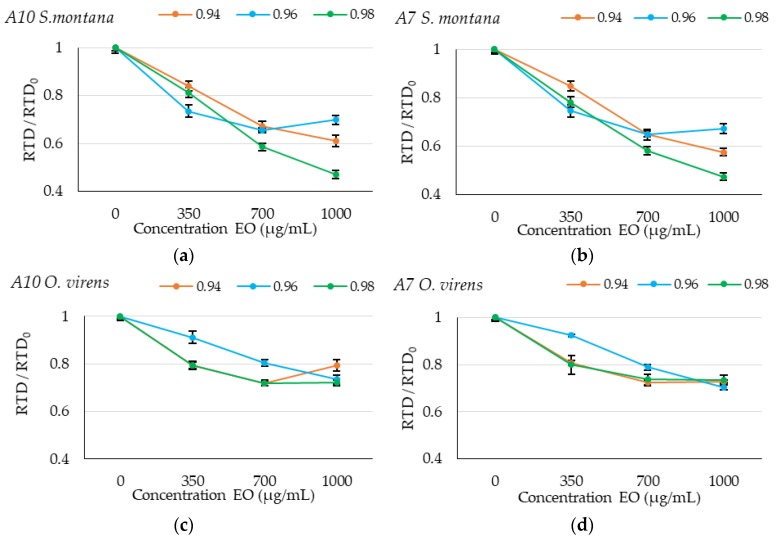
Graphical representation of relative rate to detection (RTD) (RTD/RTD_0_) at different concentrations (0, 350, 700 and 1000 µg/mL) of *Satureja montana* (SM) and *Origanum virens* (OV) essential oils; (**a**) A10 strain with SM essential oil, (**b**) A7 with SM essential oil, (**c**) A10 strain with OV essential oil and (**d**) A7 strain with OV essential oil. The different a_w_ levels studied (0.94, 0.96 and 0.96) are represented in the legend. Data represent the average of the relative RTD of 10 replicates.

In order to further study the effect of different concentrations of EOs and their interaction with environmental factors in Figure 3, we represented the rate to detection (RTD, 1/TTD) at 0.2 nm calculated in all conditions tested and normalized by the RTD_0_, which corresponds to the control without EO treatment (RTD/RTD_0_). If the effectiveness of the EOs was the same under the different environmental conditions, in these graphs, the lines should be superposed to each other. This also allows for the comparison of the efficacy between different antifungals. 

In the specific case of SM EO shown in Figure 3a,b, it can be observed that wetter conditions (0.98 a_w_) will allow higher control under the highest concentrations in comparison with the other dryer conditions tested. For OV EO, it can be observed in Figure 3c,d that there is a clear interaction at 0.96 a_w_ where the antifungal effect is decreased. 

For both *A. flavus* isolates, the presence of SM EO showed higher antifungal activity compared to OV EO treatment at all the a_w_ levels tested. 

### 2.2. Effectiveness of Satureja montana and Origanum virens Essential Oils at Different Water Activity Levels in Reducing Aflatoxin Production

The amount of aflatoxin B_1_ and B_2_ (AFB_1_ and AFB_2_) produced after 7 days of incubation at three a_w_ after the treatments with SM and OV EOs was determined, and results are shown in Table 1. 

The statistical analyses regarding AFB_1_ and AFB_2_ produced by *A. flavus* A10 strain in the presence of SM EO showed significant differences among concentrations (*p* < 0.0001), a_w_ (*p* < 0.0001) and their interaction (*p* < 0.0001). Comparing between strains, the A10 isolate was able to produce higher levels of AFB_1_ and AFB_2_. Significant reductions were achieved for both toxins when SM EO was used at 0.96 and 0.98 a_w_, with percentages of 85% and 94%, respectively, in AFB_1_ concentrations of 1000 µg/mL of SM EO. For AFB_2_, 90% and 94% reductions were observed at the same dose of SM EO. It is important to highlight the reduction in AF production found at 0.96 a_w._ *Aspergillus flavus* A10 reached very high levels of production in control assays and more than 84% of reduction was obtained even at the low dose of SM EO (350 µg/mL) tested. 

The treatment with OV EO had a lower effect on AF production. The statistical analyses of AFB_1_ for the A10 strain showed significant differences between a_w_ (*p* < 0.0001) but no effect on EO concentration (*p* = 0.3140) or the interaction of both factors (*p* = 0.3323). In the case of AFB_2_, there were significant differences among the a_w_ levels (*p* < 0.0001) and EO concentrations (*p* = 0.050), whereas the interaction between factors was not significant.

*Aspergillus flavus* A7 produced lower levels of AFB_1_ and AFB_2_ than A10. In most cases, the levels were below the detection limits. The statistical analyses regarding AFB_1_ and AFB_2_ produced in the presence of SM EO showed significant differences among the concentrations (*p* < 0.0001) and a_w_ levels (*p* < 0.0001), as well as a significant interaction between both factors (*p* < 0.0001). The OV EO treatment showed significant differences among a_w_ levels (*p* < 0.0001) and EO concentrations (*p*_B1_ < 0.0001 and *p*_B2_ = 0.0040), as well as the interaction of factors (*p*_B1_ = 0.0003 and *p*_B2_ = 0.0008).

## 3. Discussion

Environmental sustainability, as well as ensuring food safety, are important issues which have increased the search for new products that might be applied as fungicides or natural preservatives, to replace synthetic chemicals to control the growth of toxigenic species in agrifood products. It has been widely demonstrated that essential oils (EOs) could be a good alternative to reduce fungal growth and mycotoxin production by several toxigenic species [11]. However, to develop appropriate control strategies to be applied in food matrices, it is important to study fungal behavior under different environmental conditions. Several authors have reported a variation in the effectiveness of fungicide treatments under different environmental conditions, mainly temperature or humidity [9,22]. In order to establish the interaction of these compounds with environmental factors (i.e., storage or conservation conditions), in vitro ecophysiological studies are a good option since they provide accurate and rapid results. There is also a need for rapid in vitro techniques that give significant information on the range of actions of these compounds under different environmental conditions. The Microbiological Growth Analyzer, Bioscreen-C, is a fast system that allows the evaluation of the effects of these new control agents under a combination of various environmental factors [23]. Moreover, this method has been successfully applied to evaluate the growth of filamentous organisms through automated monitoring [20,24]. In addition, this system provides an inexpensive tool to simultaneously test various compounds and to establish their optimal environmental conditions to be applied. This method allows us to plan large-scale studies since it is composed of two 100-well plates, requiring a minimal volume (300–500 µL/well), and all wells can be treated independently [20]. As mentioned before, the parameter studied in this work was the time to detection (TTD, time in which fungal growth is detected at a certain biomass level), which makes the calculation independent of the experimental time [20]. The study of these parameters is a very good approximation to understand the growth of fungal colonies in a 3D space and at very low biomass levels [10].

Recent consumer trends towards safer foodstuffs, produced using sustainable and ecofriendly methods, have sparked great interest in new alternatives to traditional chemical food preservatives or synthetic fungicides [13]. In a recently published work carried out by our group, the EOs extracted from *Satureja montana* (SM) and *Origanum virens* (OV) were demonstrated to be effective to controlling *A. flavus* growth and its ability to produce aflatoxins (AF) in vitro or in maize grains when humidity was maintained at high levels [25]. Considering the influence of environmental factors on the effectiveness of EOs, the objective of the present work was to determine if they were able to control fungal growth and AF production at three different water activity (a_w_) levels. Moreover, it is known that the additional stress posed by fungicide agents may stimulate mycotoxin production as a defense reaction when environmental conditions vary [26]. Our results, which showed that under specific temperature x a_w_ combinations, the amount of toxin was increased, confirming the necessity of performing this kind of integrated experiment to test the effectiveness of antifungal compounds at different doses and in a wide range of conditions. This is especially important in the case of low-producing strains, such as the one we used (A7), which without EOs would not be considered as a problem in terms of food safety. However, AF production spikes were detected at certain conditions of a_w_ under the presence of both EOs, increasing the potential risk for consumers.

In this study, we have demonstrated that SM EO was able to retard fungal growth and reduce AF production, mainly at the highest a_w_ levels tested. The results obtained regarding the effect of SM EO on fungal growth and AF production by *A. flavus* at 0.98 a_w_ using Bioscreen C are similar to those reported in previous in vitro studies in our laboratory [25]. The most significant results in this case were observed at 0.96 a_w_, which is coincident with the conditions in which both isolates reached the highest levels of AF production. In this latter case, the levels of inhibition reached significant values in the case of fungal growth and AF production, respectively, even at the lowest dose tested (350 µg/mL). Therefore, the application of this EO might be adequate during storage when the moisture levels of the products are quite high. As mentioned above, the treatment with SM EO was not effective to significantly reduce AF production at 0.94 a_w._ However, production levels reached at this a_w_ are quite low, even at control conditions compared with other conditions, which again reveals the relevance of maintaining good storage conditions to avoid the AF contamination of agrifood products, and it would not be necessary to apply any fungicide treatment. Besides, this treatment would be applied in regions with wet weather conditions where the maintenance of these good storage practices is difficult and expensive.

Taking into account our results, the application of SM EO would be appropriate in food products with high water content and those that are frequently contaminated by AFs such as sorghum, almonds, pistachio and rice. On the contrary, the treatment of dried food matrices might not be necessary due to the inability of *A. flavus* to produce AFs in these extreme conditions.

## 4. Conclusions

Our results demonstrate that the application of *Satureja montana* essential oils might be a good option to prevent aflatoxin contamination of food products, although its effectiveness widely differed among water activity (a_w_) levels. At lower a_w_ conditions, *A. flavus* growth was significantly delayed and aflatoxin production was consistently reduced compared to other humidity conditions, but no effect was observed after essential oil treatment. This means that if it is applied in dry matrices or when good storage practices are applied, it will be an unnecessary cost for the producers. Meanwhile, application in wetter conditions will be adequate as an effective control method to guarantee low levels of aflatoxins. 

## 5. Materials and Methods

### 5.1. Microorganism and Essential Oils

#### 5.1.1. Fungal Strains

Two aflatoxin-producing strains of *A. flavus* were used (A7 and A10). They were isolated from maize and oats in different works performed in our laboratory. The correct identification of these isolates was confirmed using a species-specific PCR protocol [27]. The strains were selected due to their ability to produce aflatoxins (AFs). The strain A10 was classified as a high toxin-producing isolate (5.90 ng/µL AFB_1_ + 0.43 ng/µL AFB_2_), whereas the strain A7 was able to produce low levels of AFs (0.33 ng/µL AFB_1_ + 0.03 ng/µL AFB_2_).

The strains were maintained by regular subculturing on potato dextrose agar medium (PDA (Pronadisa, Madrid, Spain)) at 25 ± 1 °C for 5 days in the dark, and stored as a spore suspension in 15% glycerol (Panreac, Madrid, Spain) at −80 °C until required.

#### 5.1.2. Essential Oils of Plant

The essential oils (EOs) tested from *Satureja montana* L. (SM) and *Origanum virens* Hoffmanns and Link (OV) were provided by The Agricultural Research Centre of Albaladejito (Cuenca, Spain). Extraction was previously described by García-Díaz et al. (2019) [25]. Briefly, each plant species was extracted by hydrodistillation of the dried aerial parts of aromatic plants, following the methodology proposed by the European Pharmacopoeia in a Clevenger-type apparatus for 2 h. The chromatograms are shown in Appendix B.

These compounds were filtered with sterile 0.22 μm pore size filters (Fisher Scientific, Madrid, Spain) and stored at −20 °C in amber glass vials (Thermo Scientific, Madrid, Spain), until required.

### 5.2. Experimental Design

Semisolid YES (Yeast Extract Sucrose) medium (20 g/L of yeast extract, 150 g/L of sucrose, 0.5 g/L of magnesium sulfate and 0.5 g/L of agar [20]) at different water activities (a_w_) (0.94, 0.96, and 0.98) was spiked with different concentrations of the EOs. The a_w_ of the YES medium was modified by substituting water with glycerol [28].

The essentials oils of SM and OV were diluted in 5 mL of YES media to obtain final concentrations of 350, 700, and 1000 μg/mL. The control medium was supplemented by the same volume of water instead of EO.

The initial spore suspensions of each strain were prepared in sterile saline solution (9 g/L sodium chloride (Merck, Darmstadt, Germany)). After homogenizing, the spore concentrations of the solutions were measured using a Thoma counting chamber (Marienfeld, Lauda-Königshofen, Germany) and then adjusted with a sterile solution to a final concentration of 10^7^ spores per mL. 

Every medium was inoculated with 50 μL of a 10^7^ spores/mL suspension of the corresponding strain. The resulting final concentration was 10^5^ spore/mL in the YES medium for each strain, EO concentration and a_w._ A total of 48 conditions were evaluated. Ten replicates per treatment were carried out.

Three hundred µL of inoculated media, as well as noninoculated controls, were placed in 100-well honeycomb plates and incubated at 25 ± 1 °C for 7 days in the Bioscreen C Microbiological Growth Analyser (Labsystems, Helsinki, Finland).

The optical density (O.D) was automatically recorded every 30 min using a 600 nm filter over 7 days (10,080 min). The data were recorded using the software Easy Bioscreen Experiment (EZExperiment) provided by the manufacturer and then exported to a Microsoft Excel Professional 2010 (Microsoft Corporation, Washington, USA) datasheet for further analyses.

### 5.3. Aflatoxin Assessment

For each treatment and condition, 3 replicates corresponding to the content of 3 wells were transferred to 2 mL Eppendorf tubes. AF extraction was carried out with 0.8 mL of chloroform (Merck, Darmstadt, Germany), by vigorous shaking for 60 min. The mix was then centrifuged for 5 min at 5000 rpm (Centrifuge 5417 R (Eppendorf, Stevenage, UK)). The aqueous phase was decanted, and the chloroform phase was transferred to a new tube. The samples were evaporated to dryness in a miVac vacuum centrifuge (SP Scientific, Suffolk, United Kingdom), and the residues were redissolved in 500 μL methanol/water (50:50; *v*/*v*). The samples were filtered using a nylon syringe filter, 0.22 μm pore size (Minisart^®^, Sartorius Stedim, Germany), and were transferred into HPLC-FLD vials and stored at −20 °C until analysis.

The samples were analyzed by an HPLC-FLD detector (Agilent1200 series HPLC, Agilent, Cheadle, UK), coupled to a UVE photochemical derivatizer (LCTech, Obertaufkirchen, Germany). The FLD detector excitation and emission wavelengths were 330 and 460 nm, respectively. Chromatographic separations were performed on a C_18_ column ZORBAX-Eclipse Plus (4.6 × 150 cm, 3.5 µm (Agilent, Cheadle, UK)). Methanol/water/acetonitrile (30:60:10; *v*/*v*/*v*) was used as the mobile phase at a flow rate of 1 mL/min. AFG_2_, AFG_1,_ AFB_2_ and AFB_1_ were eluted at 6.5, 7.6, 8.7 and 10.4 min, respectively. The signals were processed by Agilent Chem-Station software (Agilent Technologies, Palo Alto, CA, USA). AFs were quantified on the basis of the HPLC fluorimetric response compared to a range of mycotoxin standards supplied by Romer Labs (Romer Labs, Runcorn, UK). The limit of detection (LOD) of the analysis was 0.52 ng for AFB_1_ and AFG_1,_ and 0.06 ng for AFB_2_ and AFG_2_, based on a signal to noise ratio of 3:1.

### 5.4. Data Analysis

The raw datasets obtained from the Bioscreen C were subjected to two further steps before analysis. First of all, the average of the first 5 measurements (180 min) for each well was calculated, and the average was subtracted from all subsequent measurements in order to correct the different signal backgrounds. Subsequently, the time to detection (TTD) for 0.2 nm of O.D was obtained using a Microsoft Excel template (kindly provided by Dr. R. Lambert), which used linear interpolation between successive O.D readings [29].

Once the TTDs were obtained, analysis of variance (ANOVA) was performed using the different concentrations of EOs (0, 350, 700 and 1000 μg/mL) and a_w_ (0.94, 0.96 and 0.98) as independent variables to evaluate the effect of SM and OV essential oils on the fungal growth of A7 and A10 strains of *A. flavus*. In all cases, statistical analysis was performed independently for each EO and isolate. Because of the lack of normality of the AF production datasets, ANOVA analysis was performed using a log-transformed dataset. The mean comparisons for each independent variable (EO concentration and a_w_) were done using Tukey’s HSD. The statistical package JMP 8 (SAS Institute Inc., 2008; Cary, NC, USA) was used in the analysis.

## Figures and Tables

**Table 1:** Aflatoxin concentrations (B_1_ and B_2_) produced by *A. flavus* isolates (A10 and A7) in the presence of different concentrations (0, 350, 700 and 1000 µg / mL) of *Satureja montana* (SM) and *Origanum virens* (OV) essential oils (EOs), under different water activity levels (0.98, 0.96 and 0.94 a_w_). Values are the means of 3 replicates ± standard errors. Means with a common letter are not significantly different (*p* > 0.05). In all cases statistical analysis was performed independently for each EO and isolate.

E.O	a_w_	μg/mL	*A. flavus* A 10		*A. flavus* A 7	
			B_1_ (µg/g agar)	B_2_ (µg/g agar)	B_1_ (µg/g agar)	B_2_ (µg/g agar)
SM	9400	0	751 ± 79 ^ab^	28 ± 2 ^a^	N.D ^a^	N.D ^a^
		350	786 ± 762 ^ab^	32 ± 34 ^a^	N.D ^a^	N.D ^a^
		700	572 ± 143 ^a^	28 ± 7 ^a^	22 ± 7 ^a^	N.D ^a^
		1000	404 ± 35 ^a^	27 ± 2 ^a^	N.D ^a^	N.D ^a^
	9600	0	58,235 ± 3061 ^d^	1856 ± 114 ^d^	56 ± 1 ^ab^	N.D ^a^
		350	9525 ± 5155 ^bc^	251 ± 136 ^ab^	77 ± 8 ^ab^	N.D ^a^
		700	5205 ± 3533 ^abc^	103 ± 69 ^ab^	358 ± 14 ^c^	5 ± 0 ^b^
		1000	8594 ± 3084 ^abc^	183 ± 74 ^ab^	5 ± 7 ^a^	N.D ^a^
	9800	0	13,633 ± 2270 ^c^	542 ± 99 ^c^	195 ± 135 ^b^	N.D ^a^
		350	8076 ± 3053 ^abc^	313 ± 113 ^bc^	N.D ^a^	N.D ^a^
		700	275 ± 27 ^a^	11 ± 3 ^a^	N.D ^a^	N.D ^a^
		1000	786 ± 285 ^ab^	31 ± 12 ^a^	N.D ^a^	N.D ^a^
OV	9400	0	1614 ± 34 ^ab^	91 ± 1 ^ab^	132 ± 8 ^bc^	3 ± 0 ^bc^
		350	1496 ± 153 ^ab^	82 ± 9 ^a^	98 ± 9 ^abc^	2 ± 0 ^abc^
		700	911 ± 72 ^ab^	49 ± 6 ^a^	60 ± 2 ^abc^	N.D ^a^
		1000	370 ± 59 ^a^	25 ± 4 ^a^	N.D ^a^	N.D ^a^
	9600	0	14,136 ± 10,836 ^abcd^	342 ± 254 ^abc^	175 ± 5 ^cd^	2 ± 0 ^abc^
		350	24,284 ± 3092 ^d^	664 ± 124 ^c^	286 ± 71 ^d^	4 ± 1 ^c^
		700	20,737 ± 9082 ^cd^	504 ± 222 ^c^	278 ± 38 ^d^	4 ± 1 ^c^
		1000	15,866 ± 1696 ^bcd^	339 ± 54 ^abc^	68 ± 74 ^abc^	1 ± 2 ^ab^
	9800	0	13,671 ± 831 ^abcd^	551 ± 32 ^abc^	17 ± 1 ^ab^	N.D ^a^
		350	11,751 ± 876 ^abcd^	472 ± 35 ^abc^	18 ± 2 ^ab^	N.D ^a^
		700	6827 ± 766 ^abc^	296 ± 36 ^abc^	N.D ^a^	N.D ^a^
		1000	7315 ± 755 ^abc^	321 ± 22 ^abc^	5 ± 7 ^a^	N.D ^a^

N.D: Not detected (values below detection limits).

**Figure A1:**
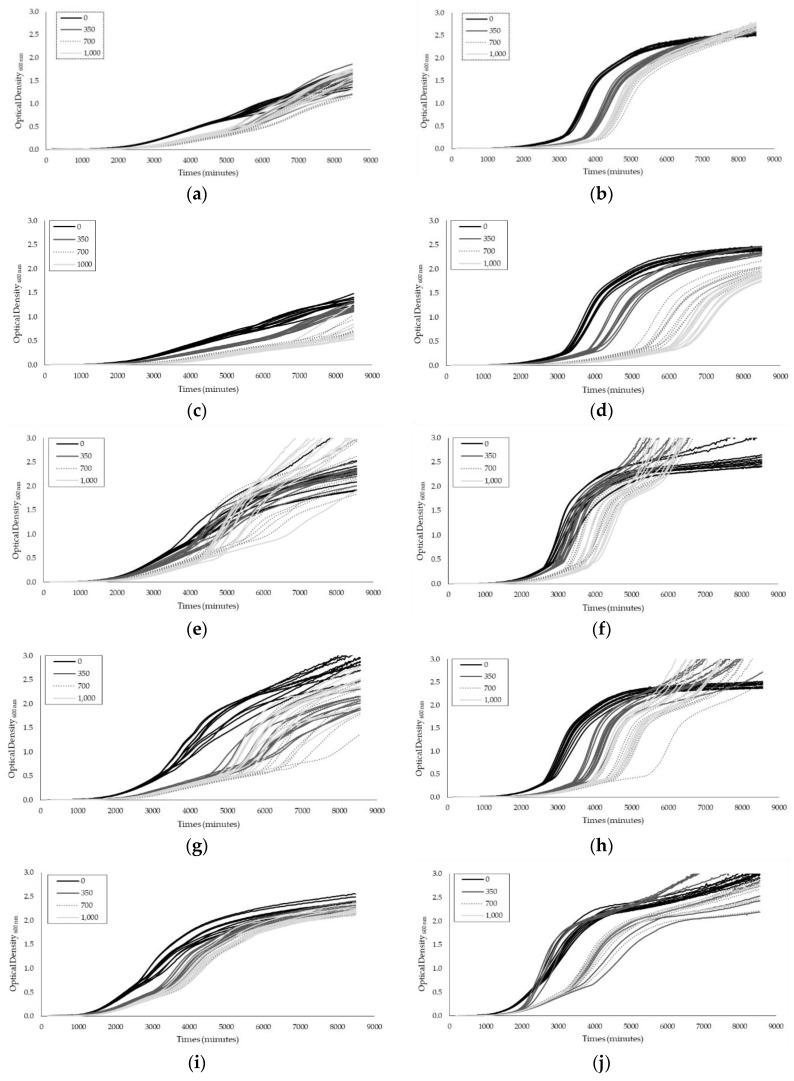

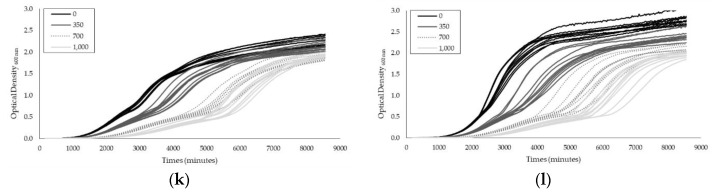
(**a**) Growth curve A10 strain at 0.94 a_w_ in OV, (**b**) Growth curve A7 strain at 0.94 a_w_ in OV, (**c**) Growth curve A10 strain at 0.94 a_w_ in SM, (**d**) Growth curve A7 strain at 0.94 a_w_ in SM, (**e**) Growth curve A10 strain at 0.96 a_w_ in OV, (**f**) Growth curve A7 strain at 0.96 a_w_ in OV, (**g**) Growth curve A10 strain at 0.96 a_w_ in SM, (**h**) Growth curve A7 strain at 0.96 a_w_ in SM, (**i**) Growth curve A10 strain at 0.98 a_w_ in OV, (**j**) Growth curve A7 strain at 0.98 a_w_ in OV, (**k**) Growth curve A10 strain at 0.98 a_w_ in SM, (**l**) Growth curve A7 strain at 0.98 a_w_ in SM.

## Appendix A

The growth curve obtained in Bioscreen-C for two strains of *A. flavus* (A10 and A7), under the different essential oil concentrations (0, 350, 700 and 1000 µL) of *Satureja montana* (SM) and *Origanum virens* (OV), to the three water activities (0.94, 0.96 and 0.98 a_w_) tested.

## Appendix B

**Table A1:** Chromatograms of *Origanum virens* and *Satureja montana* essential oils.

Time (minutes)	Compound	Area (%)	
		*Satureja montana*	*Origanum virens*
11.03	tricyclene	ND	1.7
11.04	alpha-thujene	2.0	ND
11.39	alpha-pinene	1.5	0.7
12.01	Canfeno	0.6	0.2
12.63	Sabineno	0.9	0.6
12.98	beta-pineno+Myrcene	5.7	2.4
13.81	delta-3-carene	0.5	0.4
14.18	alpha-terpinene	3.2	4.5
14.49	para cymene	12.6	3.6
14.64	Limoneno	1.6	0.4
15.70	gamma-terpinene	22.5	46.0
16.18	cis sabinene hydrate	0.5	0.3
17.02	linalool	1.6	0.1
20.13	borneol	1.4	0.2
20.32	terpinen-4-ol	1.0	0.5
22.16	nerol	ND	1.3
23.97	thymol	2.0	21.0
24.42	carvacrol	34.6	0.1
28.70	trans-caryophyllene	1.5	5.1
29.84	e-beta-farnesene	0.1	0.9
30.60	valencene	0.2	3.0
31.08	bicyclogermacrene	1.0	2.1

ND: not detected (values below detection limits).
